# PKC-δ isoform plays a crucial role in Tat-TLR4 signalling pathway to activate NF-κB and CXCL8 production

**DOI:** 10.1038/s41598-017-02468-8

**Published:** 2017-05-24

**Authors:** Manutea Serrero, Rémi Planès, Elmostafa Bahraoui

**Affiliations:** 1 CPTP, U1043, INSERM/CNRS/UPS, Toulouse, France; 2 Université Paul Sabatier Toulouse 3, Toulouse, France

## Abstract

HIV-1 Tat protein induces the production of CXCL8 chemokine in a TLR4/MD2 and PKC dependent manner. The objective of this study was to understand whether these two pathways were distinct or constituted a single common pathway, and to determine the nature of the PKC isoforms involved and their interrelation with the activation of NF-κB and CXCL8 gene product expression. Here, we show that Tat-induced CXCL8 production is essentially dependent on the activation of PKC delta isoform, as shown a) by the capacity of PKC delta dominant negative (DN), and Rottlerin, a selective PKC delta pharmacological inhibitor, to inhibit Tat-induced CXCL8 production and b) by the ability of the constitutively active (CAT) isoform of PKC delta to induce CXCL8 production in a HEK cell line in the absence of Tat stimulation. The finding that comparable amounts of CXCL8 were produced following stimulation with either Tat protein, PKC-delta CAT transfection, or both, argue for the implication of one common pathway where PKC delta is activated downstream of TLR4 recruitment and leads to the activation of NF-κB. Altogether, our results underline the crucial role of PKC delta isoform in activating gene expression of CXCL8, a cytokine largely implicated in the physiopathology of HIV-1 infection.

## Introduction

Human Immunodeficiency Virus type-1 (HIV-1) infection is associated with large and continuous production of pro-inflammatory cytokines/chemokines including TNF-α, IL-1β, IFN-α, IL-6, and CXCL8 (previously named IL-8)^1–7^. This pro-inflammatory state contributes to the establishment of a chronic hyperactivation of the immune system leading not only to its weakening^8–12^ but also to the development of several other disorders including neurological and cardiovascular pathologies^13^. In HIV-1 infection, the persistent activation of the immune system seems to be essentially initiated by HIV-1 infection following the recruitment and activation of various Pattern Recognition Receptors (PRR) by the proteic (gp120 via TLR2 and TLR4^14, 15^, gp41 via TLR2/1, p24 via TLR2/6^16^, Tat via TLR4^17^, VpR via TLR4^6^) and nucleic acid (the uridine rich ssRNA via TLR7/8, and the double stranded DNA via c-GAS^18^) viral components^19^. Secreted pro-inflammatory cytokines/chemokines also participate in the stimulation of viral replication, leading to the rapid depletion of activated CD4+ T cells in the gut-associated lymphoid tissue (GALT), within the two first weeks post-infection^20, 21^.

Furthermore, the persistence of HIV-1 replication and the inflammatory state in the GALT contribute to the alteration of the gastrointestinal epithelial barrier, thus leading to microbial translocation from the intestinal lumen to the circulating blood^22^. Accordingly, Brenchley *et al*.^22^ have shown increased levels of lipopolysaccharide in humans during chronic HIV-1 infection. This effect has also been confirmed in the SIV/macaque model^22^. In addition to LPS, relatively high levels of other microbial products, such as lipoteichoic acid (LTA), and bacterial DNA, are found to be present in the blood and also in other compartments, such as the peripheral lymph nodes and liver. These translocated bacterial PAMPs (Pathogen Associated Molecular Patterns) seem to act more strongly and continuously as a secondary stimulation signal, maintaining a persistent chronic immune activation and inflammatory state by activating various PRR such as TLR4 by LPS, TLR2 by LTA and TLR9 by DNA, in various organ tissues^23^.

The crucial role of immune activation and inflammation in AIDS development is largely documented by the data of animal-model experiments describing the evolution or absence of evolution of the AIDS disease in non-human primates that are not natural SIV hosts, such as Asian macaques, and non-human primate natural SIV hosts, such as African Green Monkeys and Sooty Mangabeys, respectively^24^. Among the cytokines/chemokines produced in the course of HIV-1 infection, CXCL8 has been shown to be important in HIV-1 pathogenesis development^25^. CXCL8 belongs to the CXC chemokine family. Its gene product is translated as a propolypeptide precursor of 99 amino acids that is subsequently cleaved to give mature CXCL8 chemokines of 72 amino acid polypeptide in immune cells and a 77 amino acid polypeptide in non-immune cells^26^. CXCL8 is produced by a variety of immune and non-immune cells including neutrophils, T cells, monocytes/macrophages, fibroblasts, epithelial cells, microglia, astrocytes and various cancer cells^27^.

CXCL8 mediates its action by interacting with two types of CXC receptors termed CXCR1 and CXCR2^28^. These receptors, members of the G protein-coupled receptor family, initially identified on neutrophils, are also present on the surface of other cells, including monocytes, T cells, astrocytes and microglia. CXCL8-CXCR1/CXCR2 interactions result in the activation of signalling pathways leading to the activation of several biological functions including chemotaxis, angiogenesis and proliferation^26, 29^. Elevated levels of CXCL8 have been found in HIV-1 infected patients, especially in the serum^30^ and cerebrospinal fluid (CSF)^25^ and CXCL8 chemokine is considered as a crucial marker to predict disease progression and AIDS related-mortality^31^. HIV-1 induces CXCL8 production via at least two mechanisms, directly via its viral components such as gp120, Nef, Vpr and Tat (for review see ref. 25) and also indirectly via IL-1β and TNF-α^32^, two proinflammatory cytokines also produced by HIV-1 infection. In this study, we focused on understanding the molecular signalling pathway activated by Tat protein to induce CXCL8.

The HIV-1 Tat gene encodes a 10–12 kDa polypeptide of 86 to 101 amino acids depending on the origin of the viral isolate. However, beside its crucial transactivating activity essential for the viral replication^33^, additional pleiotropic pathogenic and immunosuppressive activities have also been described for Tat protein^34–38^. Therefore, this protein is considered by several groups not only as a target for the development of pharmacological drugs but also as a potential vaccine candidate^39^. Recently, it was reported that immunization against Tat protein in HIV-1 infected patients under antiretroviral therapy is associated with immune system restoration and the generation of cross-clade neutralizing antibodies^40^. Interestingly, despite the absence of a signalling peptide, Tat protein is secreted by infected cells^41^ and can act on other cells, whether they are infected or not^42–44^. Tat protein is found at nM levels in the serum of HIV-1 infected patients^41, 45^. However, it can be reasonably assumed that this quantification is underestimated, due to the proportion of Tat proteins already adsorbed on the surface of cell membranes, essentially via heparan sulfates^46^, and the fact that this concentration can be much larger near the lymphoid organs and in the vicinity of infected cells.

Recently, our group has shown that HIV-1 Tat protein is able to interact physically and with high affinity (K_0.5_ = 5.10^−9^ M) with TLR4-MD2-CD14 complex to induce anti-inflammatory IL-10 cytokine and pro-inflammatory cytokines including TNF-α, CXCL8, and IL-6^17, 47^. Furthermore, we have shown that the induction of these chemokines/cytokines is totally dependent on the activation of the TLR4 pathway as demonstrated by the capacity of Tat protein to induce the production of CXCL8 in CD14-MD2-TLR4 stably transfected HEK 293-cells (HEK293-TLR4) but not in control plasmid-transfected cells (HEK-293 null), and by the ability of anti-TLR4 antibodies to block Tat-induced CXCL8 production^47^. In parallel, we have also demonstrated that HIV-1 Tat protein induces IL-10 production by human monocytes in a PKC dependent manner^48, 49^. We showed that, among the eight PKC isoforms expressed in human monocytes, Tat protein was able to activate PKC-α, PKC-βII, PKC-δ and PKC-ε isoforms^49^.

Considering the crucial role of Tat in TLR4 activation and the essential role of the PKC pathway in Tat-induced cytokine production, the objective of the present study was to: (i) understand the interrelationship between TLR4 and PKC to lead to the induction of CXCL8 production, (ii) determine the nature of the PKC isoforms involved in Tat-induced CXCL8 production, and (iii) investigate the role of TLR4 and the PKC pathway in NF-κB activation.

## Results

### TLR4 and PKC pathways are essential for HIV-1 Tat protein to induce CXCL8 production

**Figure 1:**
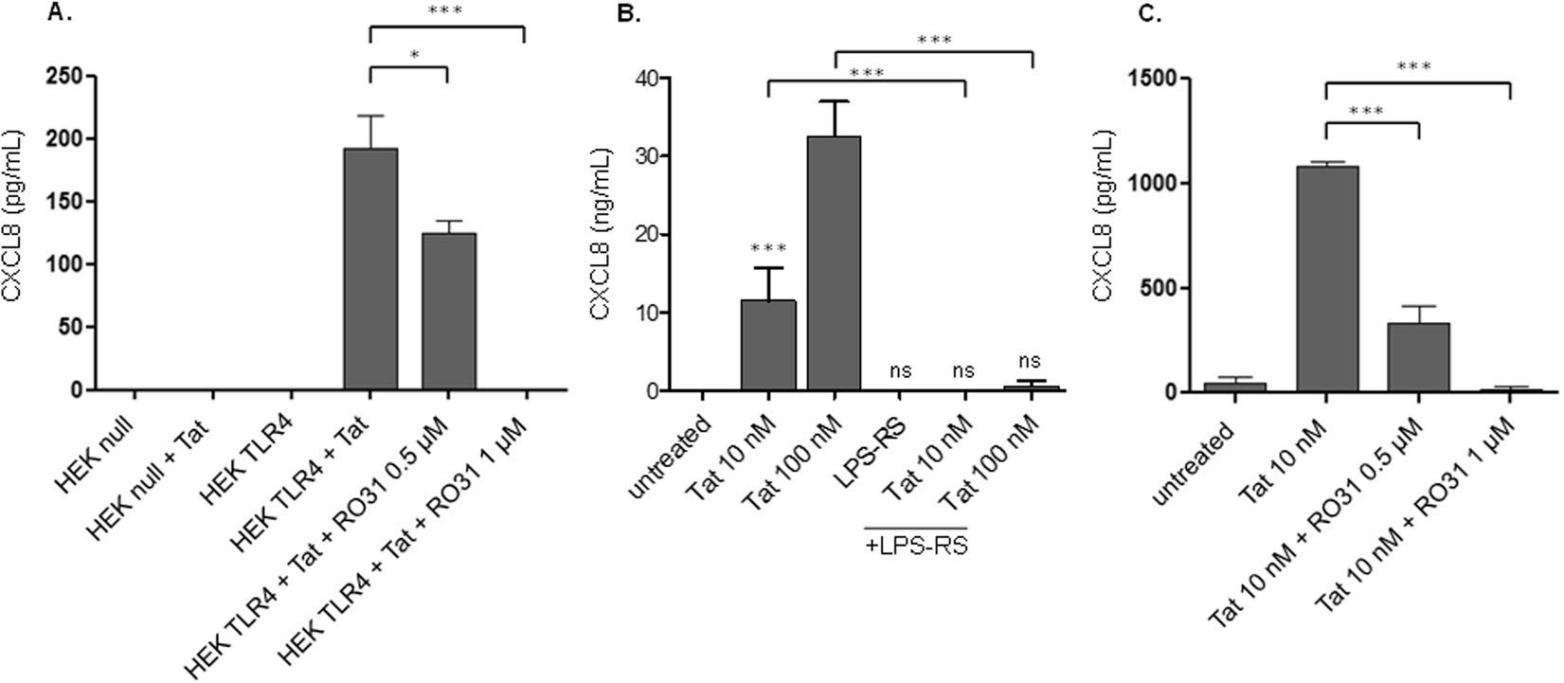
Implication of TLR4 and PKC pathways in Tat-induced CXCL8. (**A**) HEK null or HEK-TLR4 cell lines (10^6^/well) were incubated for 60 min with RO-31 8220 at 0.5 or 1 µM, a non-cytotoxic concentrations, before treatment with HIV-1 Tat protein (10 nM). (**B**) Monocyte-derived dendritic cells were incubated with Tat protein (10–100 nM) in the presence or absence of TLR4 antagonist LPS-RS (1 µg/ml). After 24 hours of incubation, cell supernatants were collected and CXCL8 production was quantified in cell supernatants by ELISA. (**C**) Primary human monocytes were treated in the same condition as in (**A**). After 24 hours of incubation, cell supernatants were collected and CXCL8 production was quantified in cell supernatants by ELISA. The data represent means and standard deviation (SD) of three independent experiments. Statistical significance comparing different group were analysed with one-way ANOVA followed with a Bonferroni post tests and are denoted with * for p < 0.05, ***p < 0.001. A black line indicates the compared bars.

In line with our previous study^50^, we demonstrated that the HEK 293 cell line, transfected with TLR4 in association with its cofactors, CD14 and MD2 (HEK-CD14-MD2-TLR4) produced CXCL8 cytokine following treatment with HIV-1 Tat protein, whereas cells transfected with the empty vector (pUNO control plasmid), HEK-null did not, thus demonstrating that the production of CXCL8 cytokine is dependent on the activation of TLR4-MD2-CD14 by HIV-1 Tat protein (Fig. 1A). In addition, we showed that Tat protein induced CXCL8 production in primary human monocytes derived dendritic cells which could be inhibited by TLR4 antagonist, using LPS-RS (from *R Sphaeroides*) (Fig. 1B). Furthermore, we showed that Tat-induced CXCL8 production was also dependent on the PKC pathway, as demonstrated by the capacity of RO31-8220, a cell-permeable pharmacological inhibitor of classical and novel PKC, to inhibit the production of CXCL8 - partially at 0.5 and totally at 1 µM - in both HEK-TLR4 and primary human monocytes (Fig. 1A and C respectively). Altogether, these data underlined the crucial roles of both TLR4 and the PKC pathway in Tat-induced CXCL8 production.

### Nature of the PKC isoforms involved in Tat-induced CXCL8 production

To further characterize the role of the PKC pathway, among the different PKC isoforms, we analysed the ones that were involved in Tat-induced CXCL8 production and further investigated their role downstream of Tat-TLR4/MD2-CD14 activation.

**Figure 2:**
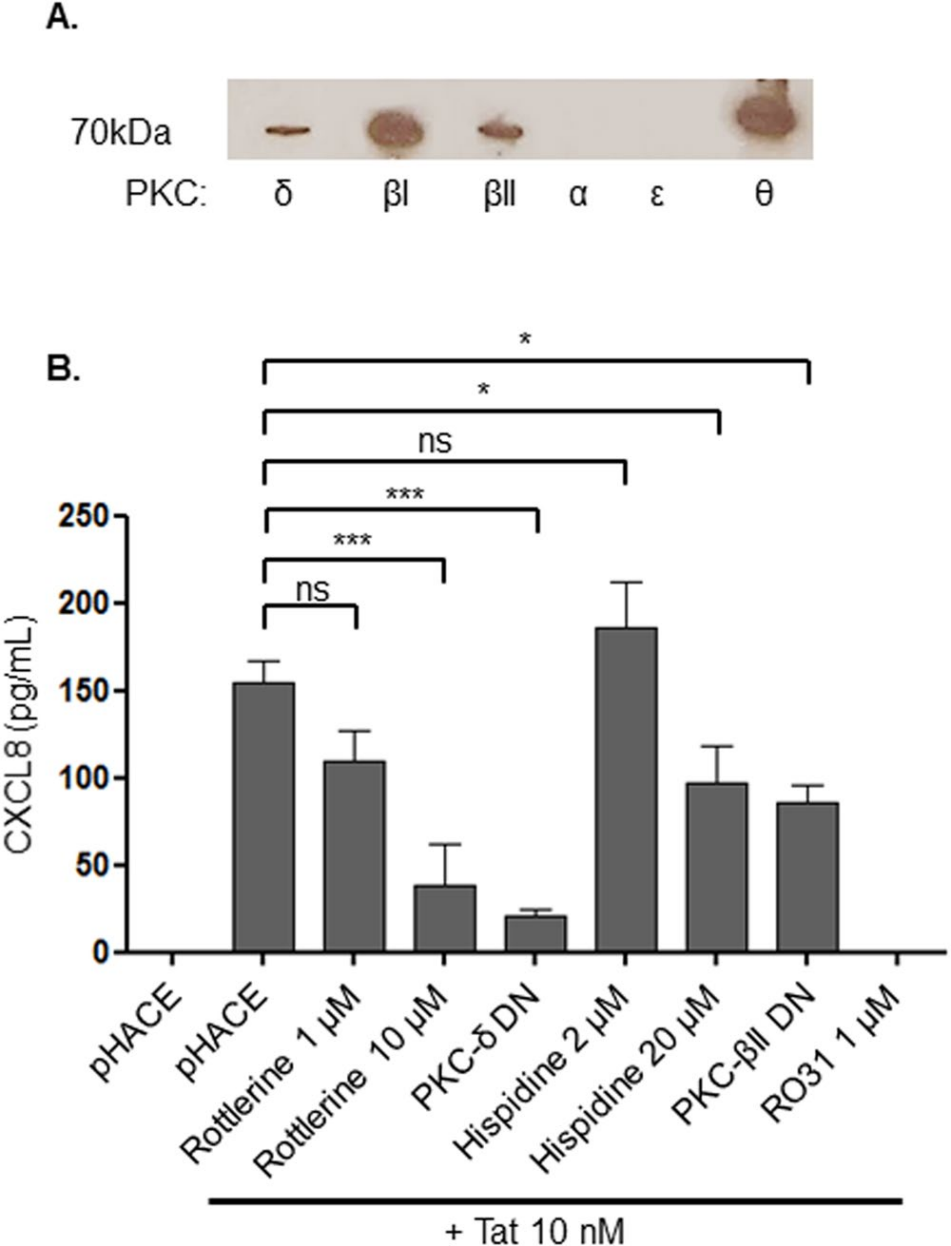
PKC-δ is essential for Tat-induced CXCL8 production. (**A**) HEK cells (10^7^ cells) were lysed and then equal amounts of protein (20 μg) were analysed by SDS-PAGE and western blot by using antibodies specific for each isoform of PKC. These results are representative from three independent experiments. (**B**) HEK-TLR4 cell lines (10^6^/well) were transfected with 0.5 µg of the empty vector pHACE, or vector encoding for PKC-βII DN or PKC-δ DN for 24 h or incubated for 60 min with RO-31 8220, a total PKC inhibitor, Rottlerin, a PKC-δ inhibitor or Hispidin a PKC-βII inhibitor before cells were treatment with HIV-1 Tat (10 nM). All chemical inhibitors were previously tested, by the trypan blue dye exclusion assay for the absence of cytotoxic effect at the concentration used (data not shown). After 24 hours of incubation, cell supernatant was collected and CXCL8 production was quantified by ELISA. The data represent means and standard deviation (SD) of three independent experiments. Statistical significance comparing different group were analysed with one-way ANOVA followed with a Bonferroni post tests and are denoted with * for p < 0.05, ***p < 0.001, ns non-significant. All bars are compared to pHACE transfected cells.

The PKC family is composed of 11 isozymes (isoforms) of serine/threonine kinases that are classified into three groups on the basis of their structure and dependence on DAG and calcium^51, 52^. To investigate the PKC isoforms implicated in Tat-induced CXCL8 production, two complementary approaches were used in this study. In the first set of experiments, western-blotting analysis was used to screen the different PKC isoforms that were expressed in HEK 293 cells. To this end, HEK 293 cell lysate proteins were separated by SDS-PAGES and analysed by western blot. The PKC isoforms were detected by using antibodies specific to each isoform. The results presented in Fig. 2A and Supplementary Fig. 1 show the expression of PKC-δ, -βI, -βII and -θ, while PKC-α and -ε isoforms were not detected (Fig. 2A). Furthermore, in a previously published work^49^ we showed that among the four PKC isoforms (PKC-α, -βII, -δ and -ε) that can be activated by Tat, only PKC-βII and -δ isoforms were crucial for the production of IL-10 in human monocytes following their stimulation by Tat^49^. Based on these acquired data, we further investigated the role of PKC-βII and -δ isoforms in Tat-induced CXCL8 production by testing the capacity of PKC-δ and PKC-βII dominant negative (DN) expression vectors to inhibit the production of CXCL8 in HEK-TLR4 cells. For this purpose, HEK-TLR4 cells were transfected with pHACE plasmids encoding for DN form of either of PKC-βII or -δ. Twenty–four hours after the transfection, the cells were stimulated with Tat (10 nM) and the production of CXCL8 in cell supernatants was quantified by ELISA 24 h post-Tat treatment. The results showed a strong inhibition of Tat-induced CXCL8 in the presence of the DN forms of PKC-δ (88%) and a moderate inhibition with the DN form of PKC-βII (45%). This result underlines the predominant importance of PKC-δ isoform (Fig. 2B). In agreement with the crucial role of PKC-δ in the signalling pathway in question, pre-incubation of HEK-TLR4 cells with Rottlerin, a pharmacological inhibitor of PKC-δ, inhibited Tat-induced CXCL8 by about 76%, while Hispidin, a chemical inhibitor of PKC-β inhibited about 38% of Tat-induced CXCL8 production, at the non-cytotoxic concentrations of 10 µM and 20 µM respectively (Fig. 2B).

### PKC δ but not PKC βII plays a crucial role in the stimulation of CXCL8 production

In order to investigate the role of these two PKC isoforms in the control of CXCL8 production, we chose to bypass the upstream steps of Tat-TLR4 signalling pathway activation by delivering, in the absence of Tat treatment, either PKC δ or βII isoforms directly as Wt (wild type), DN (dominant negative) or CAT (constitutively active) into HEK-null or HEK-TLR4 cell lines and to test their capacities to stimulate the production of CXCL8.

**Figure 3:**
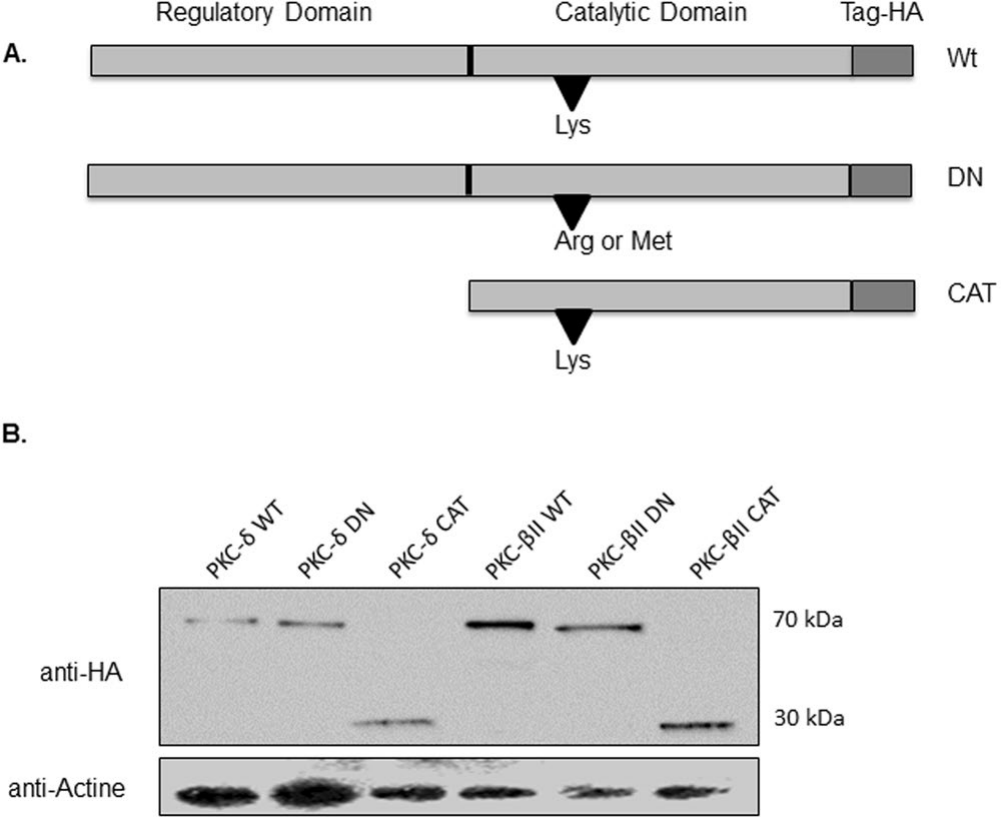
Control of PKC δ and βII expression. (**A**) schematic representation of PKC protein used in this study in either Wt, CAT of DN form. (**B**) HEK 293 T cell (10^6^ cells) were lysed and then equal amounts of protein (20 μg) were analysed by SDS-PAGE and western blot by using antibodies specific for HA-tag. Detection of Actine was used as a loading control. These results are representative of three independent experiments.

**Figure 4:**
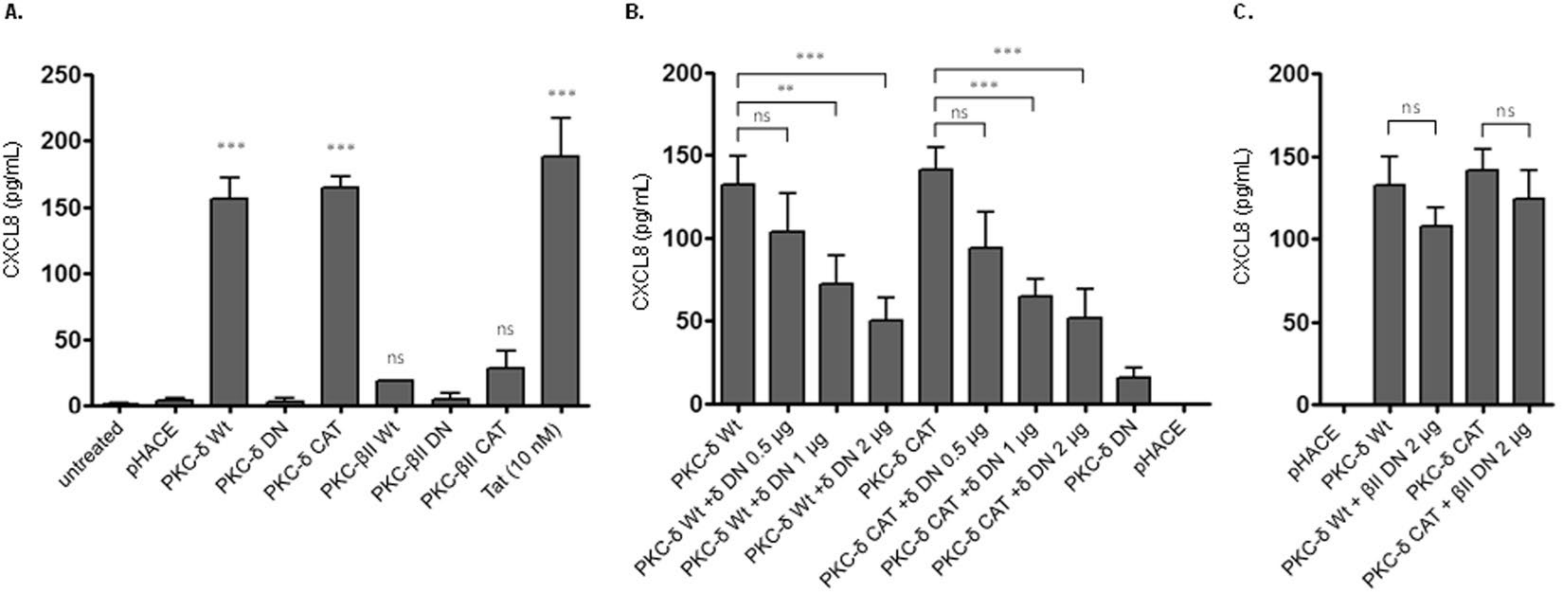
PKC-δ activation is sufficient to induce CXCL8 production. (**A**) HEK cells (10^6^ cells) were transfected with 0.5 µg of the different plasmid construction (as inducated) for 48 hours. Medium was renew 24 h post-transfection. After 24 h of incubation in the presence or absence of Tat protein (10 nM) cell supernatants were collected and CXCL8 production was quantified in by ELISA. The data represent means and standard deviation (SD) of three independent experiments. (**B,C**) HEK cells (10^6^ cells) were transfected with 0.5 µg of Wt or CAT PKC-δ in the presence of increasing amount of DN PKC-δ (0.5–2 µg) in (**B**) or 2 µg of PKC-βII DN in (**C**). After 48 hours of incubation cell supernatant was collected and CXCL8 production was quantified by ELISA. The data represent means and standard deviation (SD) of three independent experiments. Statistical significance comparing different group were analysed with one-way ANOVA followed with a Bonferroni post tests and are denoted with * for p < 0.05, **p < 0.01, ***p < 0.001, ns non-significant. Comparisons are indicated by a black line above the bars.

We first monitored the expression of HA-tagged PKC constructs. For this purpose, 24 hours post-transfection with PKC δ or βII isoforms as Wt, DN or CAT, cells were lysed and total protein extracts were analysed by SDS-PAGE and western blot. The labelling performed with anti-HA antibodies allowed the detection of gene products having a molecular weight of 70 kDa, consistent with the Wt and DN PKC isoforms, and of smaller, 30 kDa proteins consistent with CAT PKC isoforms obtained by deleting the N-terminal domain (amino-acids 1-333 and 1-328 for PKC-δ and -βII, respectively) as depicted in (Fig. 3A,B, Supplementary Fig. 2). After this validation, we tested the capacity of Wt, DN or CAT PKC-δ and -βII isoforms to induce the production of CXCL8 in HEK-TLR4, in the absence of Tat stimulation. HEK-TLR4 cells were transfected with 0.5 µg of PKC encoding or empty pHACE plasmids. Twenty four hours post-transfection, the cell culture medium was renewed, cells were cultured for an additional 24 h and CXCL8 production was quantified in cell supernatants by ELISA. In these conditions, no significant amounts of CXCL8 production were observed in the culture supernatants of cells transfected with Wt, DN or CAT PKC βII isoforms (Fig. 4A). However, expression of PKC δ Wt or CAT, but not DN, stimulated the production of CXCL8 (Fig. 4A). No CXCL8 production was observed in cells transfected with the empty vector (pHACE) or non-transfected (untreated) cells (Fig. 4A). A significant amount of CXCL8 was obtained when non-transfected HEK-TLR4 cells, as a positive control, were stimulated in the presence of Tat (10 nM) (Fig. 4A). The specificity and the essential role of PKC-δ in the induction of CXCL8 production was further investigated by testing the capacity of PKC-δ DN and PKC-βII DN plasmids to inhibit the production of CXCL8 induced by PKC Wt and CAT forms. HEK cells were co-transfected with PKC-δ Wt or CAT (0.5 µg) and increasing amounts of PKC-δ DN (0.5–2 µg). CXCL8 production was quantified in cell supernatants at 48 h post-transfection. In these conditions, a dose-dependent inhibition of PKC-δ-induced CXCL8 production was obtained with the escalating amounts of PKC-δ DN (Fig. 4B). In contrast, no inhibition was observed when the competition was performed with the same amounts of PKC-βII plasmids (Fig. 4C). The strongest inhibitions were obtained with the highest dose (2 µg) of PKC-δ DN, which inhibited CXCL8-induced PKC-δ Wt and PKC-δ CAT by 61% and 88% respectively (Fig. 4B).

### TLR4 and PKC operate through one common pathway

**Figure 5:**
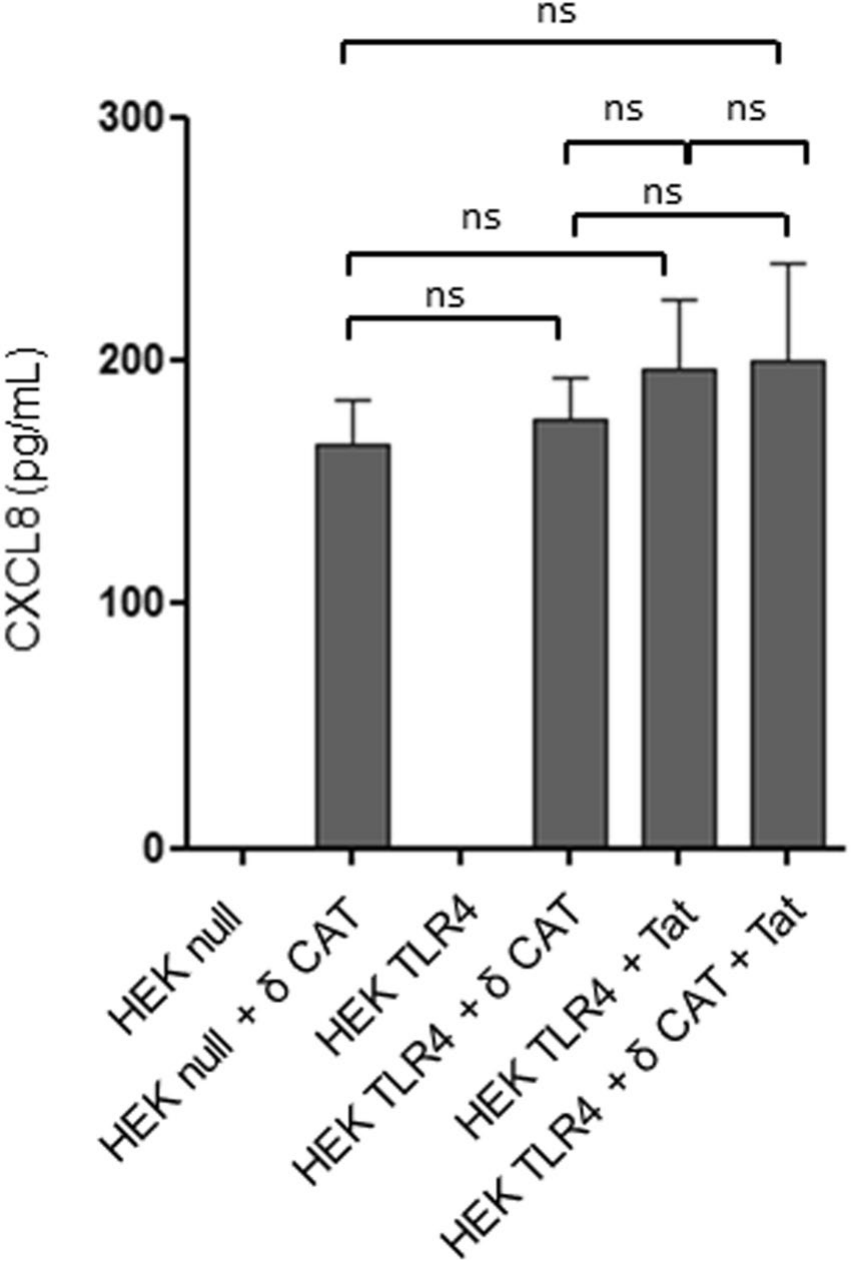
PKC-δ and Tat-TLR4 induces CXCL8 production through a common pathway. HEK cells or HEK TLR4 cells (10^6^/well) were either treated with HIV-1 Tat (10 nM), transfected with 0.5 µg of PKC-δ CAT or both. After 24 hours of incubation cell supernatants were collected and CXCL8 production was quantified by ELISA. The data represent means and standard deviation (SD) of three independent experiments. Statistical significance comparing different group were analysed with one-way ANOVA followed with a Bonferroni post tests and are denoted with ns for non-significant. Comparison is indicated by a black line above the bars.

To investigate the nature of involved pathways, we compared the magnitude of CXCL8 response, on one hand in HEK-null and HEK-TLR4 cells transfected with PKC-δ CAT and on the other hand in Tat-treated HEK-TLR4 transfected or not with PKC-δ CAT. In these conditions, the results showed not significant differences in the amount of CXCL8 production induced following PKC-δ CAT transfection, Tat treatment or both (Fig. 5), thus, suggesting that TLR4 and PKC operate through one common pathway and underlining the major involvement of the PKC-δ isoform. Together, these results indicate that downstream of Tat-TLR4/MD2 interaction, PKC-δ seems to play a pivotal role in the induction of CXCL8 production.

### PKC-delta activates NF-κB to induce CXCL8 production

Considering the implication of NF-κB transcription factor in the control of cytokine gene expression, we analysed the relationship between the constitutively active PKC-δ and NF-κB activation. Three complementary approaches were used.

**Figure 6:**
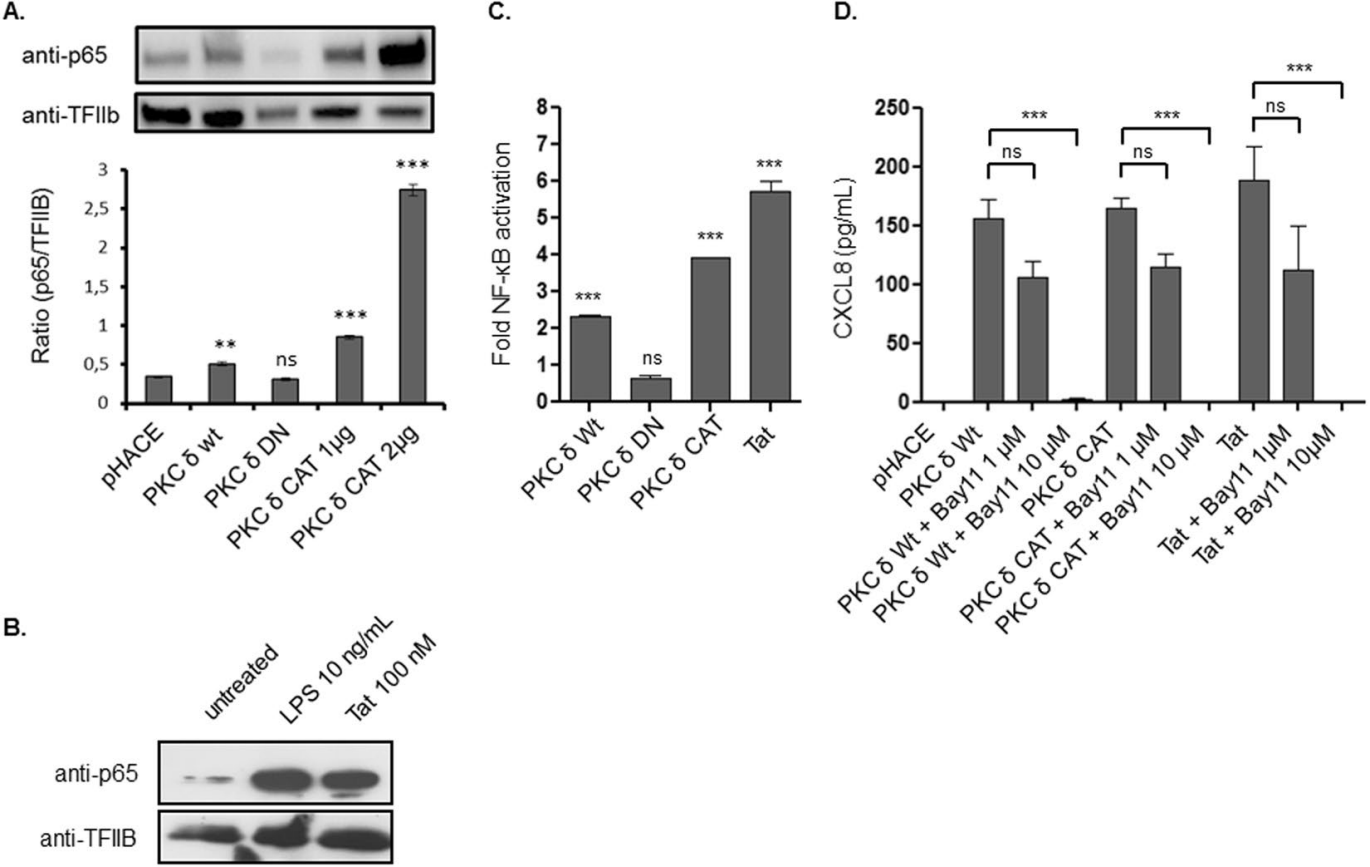
PKC-δ induces CXCL8 production via NF-κB pathway. (**A**) HEK cells (10^6^ cells/well) were transfected with 1 µg of plasmid pHACE control, or encoding for PKC-δ Wt or DN, or 1 to 2 µg of PKC-δ CAT. After 24 hours, cells were lysed and nuclear and cytoplasmic fractions were prepared. The translocation of NF-κB was monitored by analyzing p65 subunit of NF-κB in the nuclear fraction of the cells. Detection of the transcription factor TFIIB was used as a loading control. Quantification of the band obtained from 3 independent experiments was performed using ImageJ software. Data represent NF-κB nuclear expression normalized to TFIIB. (**B**) HEK-TLR4-CD14-MD2 cell lines (2 × 10^6^ cells) were left untreated or treated with Tat (100 nM) or LPS (10 ng/ml). After 60 min, cells were lysed and nuclear extracts were analysed by SDS-PAGE and western blot using anti-p65 or anti-TFIIb antibodies. (**C**) HEK-Blue™ TLR4 cells (10^6^ cells) were transfected with 0.5 µg of plasmids encoding for of PKC-δ Wt, CAT, or DN for 48 h or treated with Tat protein (10 nM) for 24 h. SEAP activity was quantified in cells supernatant using colorimetric assay. Data represents fold NF-kB activation compared to empty plasmid “pHACE” transfected cells. (**D**) HEK TLR4 cells (10^6^ cells) were pretreated with 1 or 10 µM of NF-κB inhibitor (Bay11-7280) 24 hours before transfection with 0.5 µg of PKC-δ Wt or CAT plasmids, or 1 hour before Tat treatment (10 nM). After 24 hours of incubation cell supernatants were collected and CXCL8 production was quantified by ELISA. Statistical significance comparing different group were analysed with one-way ANOVA followed with a Bonferroni post tests and are denoted with ns for non-significant. The data are compared to negative pHACE control, otherwise the comparison is indicated by a black line above the bars.

In the first approach we tested the effect of PKC-δ CAT to activate the nuclear translocation of p65. To this end HEK cell lines were transfected with PKC-δ Wt, DN or CAT plasmids. 24 h post-transfection, nuclear extracts were analysed by SDS-PAGE and western blot, using antibodies specific to p65, a subunit of NF-κB. The results showed a dose-dependent nuclear translocation of p65 in HEK cells transfected with PKC-δ CAT plasmids (1–2 µg) (Fig. 6A, Supplementary Fig. 3). No significant p65 nuclear translocations were observed in the non-transfected or PKC-δ DN transfected cells used as negative controls, while a mild but significant signal was observed in cells transfected with PKC-δ Wt (Fig. 6A). It is interesting to note that this signal, observed with PKC-δ Wt, is in line with the capacity of this isoform to activate CXCL8 production depicted in Fig. 4A. In addition we showed that soluble recombinant Tat protein, like LPS, the natural ligand of TLR4 are also able to activate NF-κB, as demonstrated by p65 translocation in HEK-TLR4-MD2 CD14 treated cells (Fig. 6B).

In the second approach, we investigated the capacity of PKC-δ CAT to activate the expression of SEAP reporter gene under the control of NF-kB inducible promotor. HEK-TLR4 cells stably transfected with the SEAP reporter gene under the control of NF-κB were transfected with PKC-δ Wt, DN or CAT plasmids. 24 h post-transfection, SEAP expression was analysed in cell supernatants. In these conditions, a clear increase of SEAP activity (39%) was observed in cells transfected with PKC-δ CAT and, at a lower level (23%), in cells transfected with PKC-δ Wt, while no significant SEAP activities were detected in non-transfected cells or in cells transfected with PKC-δ DN (Fig. 6C). In a positive control experiment, a significant increase of SEAP activity (57%) was obtained in cells treated with Tat (10 nM) (Fig. 6C). This result is in agreement with the capacity of Tat protein to activate nuclear translocation of NF-κB as shown in Fig. 6B.

In a third approach, we tested the effect of NF-κB chemical inhibitors Bay-11-7082 on CXCL8 production in response to Tat-treatment or PKC δ Wt or CAT transfection. In the presence of non-toxic concentrations (1–10 µM) of Bay11-7082, a dose-dependent inhibition of CXCL8 production was observed in HEK-TLR4 cells treated by Tat 10 nM and also in non-Tat-treated cells transfected with either PKC-δ Wt or CAT (Fig. 6D).

Altogether, our results underline the crucial role of PKC-δ, which is activated following TLR4 pathway recruitment by Tat and activates NF-κB downstream, leading to CXCL8 production. Our results also underline the importance of determining, within the panel of the activated PKC family, the isoform responsible for the establishment of a pathological state. Then, the involved PKC isoform may be used as a selective target for the development of specific inhibitor or activator ligands.

## Discussion

In this study, we have demonstrated that HIV-1 Tat protein, by recruiting the TLR4 pathway, activates PKC-δ isoform and NF-κB, leading to the production of CXCL8. This proinflammatory chemokine has been reported to be produced early in HIV-1 infection^30, 53^. Its presence and increased level are associated with the severity of AIDS development and HIV-associated neurocognitive disorders (HAND)^25^. CXCL8-associated severity has been reported to be more exacerbated in HIV-1 infected children^31^.

Among the signalling pathways activated by Tat, the PKC pathway seems to play important roles in the HIV-1 replication cycle and also in HIV-1 induced pathogenesis. Accordingly, our group have shown that PKC-δ isoform is activated following the interaction of HIV-1 R5-isolates with macrophages, and seems to be essential at early steps post–entry in the HIV-1 viral cycle^54^. The blockade of this PKC isoform with PKC inhibitors (RO31-8220 or specific antisense oligonucleotides) completely inhibited HIV-1 R5 isolate replication in macrophages^54^. PKC-θ isoform has been also reported, by another group, to be important for efficient viral replication of X4 tropic HIV-1 isolates in primary CD4-T cells^55^. In this case, the PKC-θ isoform seems to act by cooperating with Tat protein in the mechanism of HIV-1 transactivation of the HIV-1 LTR promotor^56^. The targeting of PKC pathways has gained much importance in the last few years, essentially through studies showing the ability of certain PKC agonists to activate the latent provirus. PKC activation of the latent virus is mediated mainly through the activation of NF-κB, which binds to cognate sequences in the HIV-1 LTR promotor^57^. Thus the PKC pathway could be considered as a potential therapeutic target to: (i) activate for “the shock and kill” strategy, in the aim of purging the latent HIV-1 reservoir^58^ or (ii) inhibit, in order to control the hyperactivation of the immune system and inflammation^55^. In addition to the involvement of the PKC pathway in HIV-1 pathogenesis, PKC-δ has also been reported to be implicated in several clinical diseases including diabetes^59, 60^, sepsis^61^, neurodegenerative diseases^62, 63^ and ischaemic heart diseases^64, 65^.

The PKC pathway can be activated by HIV-1 Tat^49^, Nef^1^ and gp120^66^ proteins. It is essential for Tat-induced cytokine/chemokine production including IL-1β^5^, TNF-α^67^, IL-10^68^, and MCP-1^69^. However, the precise mechanism involved in PKC activation and the nature of the PKC isoforms activated by Tat is not known. In the present study we have shown that extracellular Tat protein induces CXCL8 production in a TLR4/MD2-CD14 dependent manner that involves PKC-δ dependent activation of NF-κB.

Previous studies from our laboratory have shown that HIV-1 Tat protein interacts with high affinity with TLR4-MD2-CD14 complex^17^. This interaction leads to the production of NF-κB dependent cytokines including TNF-α, IL-10, IL-6 and CXCL8 by primary human monocytes and dendritic cells^17, 47^. Tat-TLR4 interaction has also been shown to be involved in the reactivation of endogenous retroviruses^70^ and, more recently, it has been reported that Tat synergises with LPS to exacerbate the production of pro-inflammatory cytokines^71^. The last observation is of great importance because it suggests that Tat, a protein expressed in the early phase of infection, could be considered as a key factor in the initiation of the subsequently uncontrolled hyperactivation of the immune system in HIV-infected patients. After the success obtained by HAART in the control of HIV-1 load, the second challenge to be taken up is the development of new therapies capable of controlling the persistent state of chronic activation of the immune system. One potential strategy to reach this objective could be to target specific key selective steps of signalling pathways involved in the production of proinflammatory cytokines/chemokines. Considering the major role of PKC pathways in the control of cytokine/chemokine gene expression^72^, we decided to determine the nature of the PKC isoforms implicated in the production of CXCL8, one of the key proinflammatory and chemoattractant chemokines produced early after HIV-1 infection^53^.

The involvement of the PKC pathway in Tat-induced CXCL8 production was first demonstrated by the capacity of RO-31 8220, a chemical inhibitor of PKC, to inhibit Tat-induced CXCL8 production in both primary human monocytes and HEK TLR4-MD2-CD14 cell lines. Previous studies by our group have shown that, among the 11 PKC isoforms, eight are expressed in primary human monocytes (PKC-α, βI, βII, δ, ε, η, ζ, μ). By analysing the PKC translocation from the cytoplasm (inactive form) to the membrane compartments (active form) after Tat stimulation, we have shown that PKC-α, -βI, -βII, -δ and -ε are activated by extracellular Tat^49^. Moreover, thanks to the use of different selective PKC inhibitors we have shown that PKC -βII and δ isozymes are essential for the activation of IL-10 production in human monocytes following stimulation by HIV-1 Tat protein. Others have shown that Tat activates PKC-θ in Jurkat cells, an isoform of PKC that is predominant in T cells^56^. Using the same approaches, our aim here was to decipher the mechanism of Tat-induced CXCL8 production. Our analysis of the panel of PKC expressed in HEK-293-cell lines revealed the expression of PKC-βI, -βII, -δ and -θ isoforms but detected no PKC-α or -ε. Because PKC-δ and PKC-βII where shown to be essential for Tat-induced cytokine in human monocytes, we focused on the roles of these two isoforms in the signalling pathways leading to CXCL8 gene expression. We demonstrated that PKC-δ DN and PKC-βII DN over-expression and the treatment with specific selective inhibitors of PKC-δ and PKC-βII both affected Tat-induced CXCL8 production. However, PKC-δ blockade showed the most potent inhibition of Tat-induced CXCL8 production, demonstrating the crucial role of this isoform in the signalling pathways activated by Tat to trigger NF-κB activation leading to CXCL8 gene product expression.

Several studies have reported interplay between PKC and TLR signalling pathways^72^. Here, we provide evidence that TLR4 and PKC-δ operate through one common pathway leading to CXCL8 production upon Tat treatment. A previous study from our group reported that anti-TLR4 antibody failed to inhibit Tat-induced PKC-δ cytoplasm/membrane-translocation in primary human macrophages^50^, which may be related to the known great abundance of this novel PKC isoform in monocytes/macrophages. In the present study, the use of HEK 298 TLR4-defective cell lines, instead of neutralizing antibodies, suggests that PKC-δ is involved in the signalling pathway leading to CXCL8 expression downstream of Tat-TLR4 activation. The specificity of the mode of action of PKC-δ in the present study is strengthened by the positive and negative controls using PKC-δ DN and PKC-βII DN, which respectively inhibited or failed to inhibit PKC-δ CAT-induced CXCL8. Interestingly, a significant, unexpected stimulation of CXCL8 production was also obtained following direct transfection of the Wt isoform of PKC-δ, which normally requires further activation to become functional. This apparent contradiction could be explained by the over-expression conditions, which may have led to spontaneous transition from the inactive to active state of a subset of PKC-δ Wt, sufficient to activate the downstream pathways leading to the production of CXCL8. Similar observations have been reported by Soh and Weinstein^73^, showing relative spontaneous activations of PKC Wt in over expression conditions, as demonstrated by the expression of significant kinase activities following transfection of the Wt isoforms of PKC-α, βI, βII, γ, δ, η, ζ, ι Wt. However, no kinase activities were observed when the corresponding DN isoforms were tested in the same kinase assay^73^.

Our finding of the crucial role of PKC-δ in the induction of CXCL8 production, via the activation of TLR4 by HIV-1 Tat protein, is in agreement with previous studies reported by Loegering *et al*., on the role of PKC-α, δ, ε and ζ in the activation of TLR signalling pathways^72^. In addition, Kubo-Murai *et al*. showed that PKC-δ was implicated in TLR signalling pathway by a mechanism involving its interaction with TIRAP/Mal, an adaptor protein for TLR2 and TLR4 to form a PKC-δ-TIRAP/Mal complex mediated by the TIR domain of TIRAP/Mal^74^.

Like Tat, several other HIV-1 proteins and nucleic acids have been reported to induce proinflammatory chemokines/cytokines including CXCL8^2^. Nef, a 27–35 kDa multifunctional protein, has been shown to induce CXCL8 by activating PI3K/PKC-ζ/NF-κB pathways in human astrocytes^1^. In parallel, the same group has shown that VpR, another HIV-1 14 kDa auxiliary protein implicated in the import of the viral preintegration complex, is also able to induce the production of CXCL8 following the recruitment of PI3K/Akt/NF-κB, and P38/JNK/MAPk-P38/NF-κB/C-EBP/AP-1^75, 76^. VpR, like HIV-1 Tat protein, has been shown to activate TLR4-Myd88/MAPk/NF-κB/C-EPB-pathways leading to the production of IL-6 in human macrophages^6^. In addition to auxiliary HIV-1 proteins, several structural proteins have also been reported for their capacity to activate TLR pathways, including the matrix p17 and the transmembrane gp41 proteins with TLR2/1, p24 core protein with TLR2/6 and the surface unit glycoprotein gp120 with TLR2 and TLR4^15, 16, 25^. It is interesting to note the dual synergistic role of HIV-1 matrix p17 protein, which in addition to its interaction with TLR2/1, acts as a viral-CXCL8 chemokine to interact directly with CXCR1, the major receptor of CXCL8, leading to monocyte chemotaxis through activation of Rho/ROCK pathways^77^. Several reports have shown that CXCL8 production is also amplified indirectly via IL-1β and TNF-α, two proinflammatory cytokines induced by HIV-1 viral proteins including Tat^32^.

In summary, the importance of the implication of Tat-TLR4-PKCδ-NF-κB-CXCL8 production is in line with the following findings: (i) Tat induces CXCL8 production in HEK 293 cells transfected with TLR4 in association with its cofactors, CD14 and MD2 but not in cells transfected with the empty vector. (ii) Tat-induced CXCL8 production in HEK TLR4-MD2-CD14 is inhibited by both PKC-δ chemical (Go82220 and rottlerin) or by the overexpression of PKC-δ DN. (iii) Shunting of Tat-TLR4 activation following the direct transfection of PKC-δ CAT, but not PKC-βII CAT, induces the activation of NF-κB and stimulates the production of CXCL8 in HEK 293 cells in the absence of stimulation by Tat protein. Thus these data demonstrate that the PKC-δ isoform bridges the gap between TLR4 activation by Tat and NF-κB activation leading to CXCL8 production.

Overall, our results support a model where PKC-δ activation is a key component of the signalling pathways leading to NF-κB activation and CXCL8 cytokine production following TLR4 activation by Tat protein. This finding underscores the ability to target selective PKC isoforms as a strategy in the treatment of associated viral and non-viral inflammatory diseases.

## Experimental Procedures

### Isolation of human monocytes

Peripheral Blood Mononuclear Cells (PBMCs) were isolated from buffy coat of healthy human donors from the EFS Toulouse Purpan, France as described previously^50^. Briefly, PBMC were isolated by centrifugation using standard Ficoll-Paque density (GE Healthcare). The blood was diluted 1:1 in phosphate-buffered saline (PBS) pre-warmed to 37 °C and carefully layered over the Ficoll-Paque gradient. The tubes were centrifuged for 25 min at 2000 rpm, at 20 °C. The cell interface layer was harvested carefully, and the cells were washed twice in PBS (for 10 min at 1200 rpm followed by 10 min at 800 rpm) and re-suspended in RPMI-1640 supplemented with 10% foetal calf serum (FCS), 1% penicillin (100 IU/mL) and streptomycin (100 µg/ml). Monocytes were separated from lymphocytes by adherence to tissue culture plastic (Beckton Dickinson). PBMC were seeded in 24 well plates (10^7^ PBMC/well). After an incubation of 1 h at 37 °C, non-adherent cells were removed by 3 washes with PBS (pre-warmed to 37 °C) and adherent cells were cultured in a complete RPMI-1640 medium.

### Human embryonic kidney cell line expressing TLR4/MD2-CD14

HEK cell line stably co-transfected with pUNO-TLR4 and pDUO2-MD2-CD14 were purchased from InvivoGen and maintained in culture in DMEM supplemented with 10% FCS, normocin (100 µg/mL), blasticidin (10 µg/ml) and hygrogold (50 µg/ml) at 37 °C, according to the manufacturer’s instructions of (InvivoGen).

### HEK-Blue TLR4 cells

HEK cell line expressing TLR4 and its cofactors MD2 and CD14 were also stably transfected with SEAP (secreted embryonic alkaline phosphatase), as reporter gene, under the control of NF-κB promoter. These cells were purchased from InvivoGen. In this model, the activation of NF-κB can be monitored by a colorimetric assay quantifying the activity of the secreted SEAP in the cell supernatants in the presence of enzyme substrate as described by the manufacturer (InvivoGen).

### Tat protein, antibodies and chemical products

Recombinant GST-Tat protein (1–101) from HIV-1 strain SF2 was produced and purified in our laboratory as previously described^48^. The level of endotoxin contamination was assessed using the Limulus amebocyte lysate assay (Bio-Sepra). Purified recombinant proteins contained less than 0.3 EU/µg LPS, the limit of detection of this test. LPS-RS (TLR4 antagonist) from *R Sphaeroides*, was purchased from InvivoGen. Anti-PKC-δ (C-17), and anti-PKC-βII (C-18) antibodies were obtained from cell Signalling. Monoclonal anti-human/mouse β-actin (clone AC-15) was obtained from Sigma-Aldrich. Mouse monoclonal anti-HA-Tag antibodies (clone HA-17) were purchased from Sigma Aldrich. Anti-human p65 and anti-human TFIIB (clone II B8) were purchased from Santa Cruz Biotechnology. Rabbit polyclonal anti-goat-HRP, mouse monoclonal anti-HRP and polyclonal swine anti-rabbit-HRP were purchased from Dako. The chemical products, Ro 31-8220, an inhibitor of classical (α, βI, βII, and γ) and novel (δ, ε, η, θ, and μ) PKC, Rottlerin, an inhibitor of PKC-δ, Hispidin, an inhibitor of PKC-βI and -βIIand Bay11-7082, the NF-κB inhibitor, were all purchased from Calbiochem.

### Expression pHACE plasmid constructions

Wild Type (Wt) PKC-βII and δ were obtained by ligating the full length ORF of these genes, previously HA-tagged at their C-terminal part, into pHACE plasmid between EcoR-I restriction sites. PKC-βII and δ, Dominant Negative (DN), were obtained by the same strategy as above, except that their ORF contained a point mutation at the ATP binding site (K371R and K376R for PKC βII and δ respectively). Constitutively active (CAT) PKC-βII and δ, were obtained by deleting the N-terminal part encoding for the amino acids 1-328 and 1-333 of PKC-βII and δ respectively.

### Preparation of cytoplasmic and nuclear protein extracts

After transfection with PKC encoding pHACE plasmids and/or treatment with HIV-1 Tat protein, the cells were harvested at different times (as indicated in figure legends) and rapidly lysed at 4 °C in 200 μl of hypotonic buffer A (Hepes 10 mM pH 7.9, KCl 10 mM, EDTA 0.1 mM, EGTA 0.1 mM, DTT 1 mM, PMSF 0.5 mM, Na3VO4 0.2 mM, NaF 0.05 mM) for 15 min. Then 12.5 μl of Nonidet P40 10% was added and the lysate was vortexed (20 s) before centrifugation (1 min; 14000 rpm; 4 °C). The supernatant corresponding to the cytoplasmic fraction was collected and proteins were quantified by Bradford assay and stored at −20 °C until use. The nucleus pellets were solubilized in 100 μl of cold sample buffer B (Hepes 20 mM pH 7.9, NaCl 0.4 M, EDTA 1 mM, EGTA 1 mM, DTT 1 mM, PMSF 1 mM, Na3VO4 0.2 mM, NaF 0.05 mM) and shaken strongly for 15 min at 4 °C. After centrifugation, nuclear proteins were collected in the supernatant, quantified by Bradford assay and stored at −20 °C until use.

### Western blot analysis

Equal amounts of proteins (10–40 μg) were subjected to 10% SDS-PAGE and the separated proteins were transferred to a nitrocellulose membrane. The membrane was blocked with 5% of non-fat milk in Tris-buffered saline with 0.05% Tween 20 (TTBS) for 1 h, then washed with TTBS, and incubated with the primary antibody overnight at 4 °C. Immunoreactive bands were detected by incubation for 1 h with the appropriate anti-primary antibodies conjugated with horseradish peroxidase (DAKO). Proteins of interest were visualized using a chemiluminescent substrate ECL (Pierce, Rockford, IL).

### Cytokine detection by ELISA

Adherent human monocytes (10^6^/well), or HEK cells (10^6^/well) were washed 3 times with PBS. Cells, previously transfected with the adequate plasmids, or treated with Tat, were then cultured in RPMI medium completed with 1% FCS. For PKC inhibition, cells were pre-incubated for 60 min with RO-31 8220, a total PKC inhibitor; Rottlerin, a PKC-δ inhibitor; or Hispidin, a PKC-βI and -βII inhibitor, 24 h post transfection with various pHACE PKC expression vectors or for 60 min before cell treatment with HIV-1 Tat (10 nM). A possible cytotoxic effect of PKC inhibitor, at the concentrations used, was evaluated by the trypan blue dye exclusion assay. In this condition, no significant cytotoxic effect was observed (viability > 90%). After 24 h of cell treatment, the cell supernatants were collected and the amount of human CXCL8 chemokine was quantified using ELISA kits from eBiosciences according to the manufacturer’s instructions. Briefly, the first monoclonal antibody was used for antigen capture overnight at 4 °C. After three washes with PBS containing 0.05% Tween 20 (wash buffer), plates were saturated by adding 250 µl of a saturating solution (diluent assay) for 1 h at room temperature. After three washes, culture supernatants (100 µl/well) were added and incubated for 2 h at room temperature. Plates were then washed three times and incubated for 1 h at room temperature with a biotinylated anti-cytokine antibody. After five washes, the bound biotinylated antibody was detected by an additional 30 min incubation with streptavidin peroxidase. After five washes, plates were incubated with the enzyme substrate (TMB). The reaction was stopped by adding 50 µl of H_2_SO_4_ (4 N) to each well. Absorbance was read at 450 nm with a wavelength correction at 570 nm. Cytokines were quantified from a standard curve generated by using various concentrations of recombinant CXCL8 cytokine. The limit of detection of this assay was 4 pg/ml.

### Cell transfection

HEK TLR4/MD2-CD14 cells, or HEK-null cells used as controls, were seeded into 24-well plates at 4.10^5^ cells per well the day before transfection. After 24 hours, cells (60–70% confluence) were transfected using a calcium phosphate transfection system.

### Statistical analyses

Statistical analysis was performed using GraphPad Prism software. All results are expressed as means +/−SD. All experiments were performed a minimum of three times. Differences in the means for the different groups were tested using one-way ANOVA followed by Bonferroni post hoc test or two-way ANOVA followed by a Bonferroni post hoc test (as indicated in figure legends). A p-value < 0.05 was considered statistically significant. Statistical significance comparing different groups is denoted with * for p < 0.05, **p < 0.01, ***p < 0.001, ns non-significant.

### Ethics statement

The use of human cells in this study was approved by the Research Ethical Committee, Haute-Garonne, France. Human Peripheral Blood Mononuclear Cells (PBMC) were isolated from buffy coat, from healthy human donors. Buffy coats were provided anonymously by the EFS (établissement français du sang, Toulouse, France). Written informed consent was obtained from each donor under EFS contract No. 21/PVNT/TOU/INSERM01/2011-0059, according, to “Decret No. 2007–1220 (articles L1243-4, R1243-61)”. The experiments were performed in accordance with the approved guidelines. All the authors concur with the submission and have no financial/commercial conflict of interest. The manuscript, which has not been submitted elsewhere, contains human studies which conform to the Guides for IRB and IACUC published by the US National Institute of Health.

## Electronic supplementary material

Supplementary informations
