# Supplementary material for: PKC-δ isoform plays a crucial role in Tat-TLR4 signalling pathway to activate NF-κB and CXCL8 production
Source: Sci Rep. 2017 May 24;7:2384. doi: 10.1038/s41598-017-02468-8 (PMC5443767; doi:10.1038/s41598-017-02468-8)
Supplement: Supplementary file 1 — Supplementary informations [file 41598_2017_2468_MOESM1_ESM.pdf]

PKC-δ isoform plays a crucial role in Tat-TLR4 signalling pathway to activate NF-κB and CXCL8 production.

Manutea Serrero [¶, #], Rémi Planès [¶, #], Elmostafa Bahraoui[¶, #, *]
[¶] CPTP, U1043, INSERM/CNRS/UPS, Toulouse, France.
[#] Université Paul Sabatier Toulouse 3, Toulouse, France.
* bahraoui@cict.fr

Supplementary Information
Supplementary Figure 1

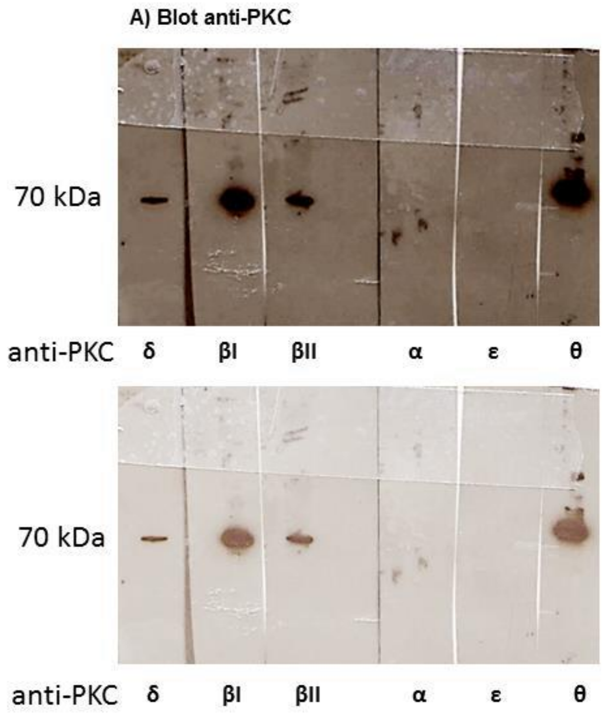

Supplementary Figure 1: Blot anti-PKC: (A) full-length blots anti-PKC related to figure 2 A. Two exposures are shown.

Supplementary Figure 2

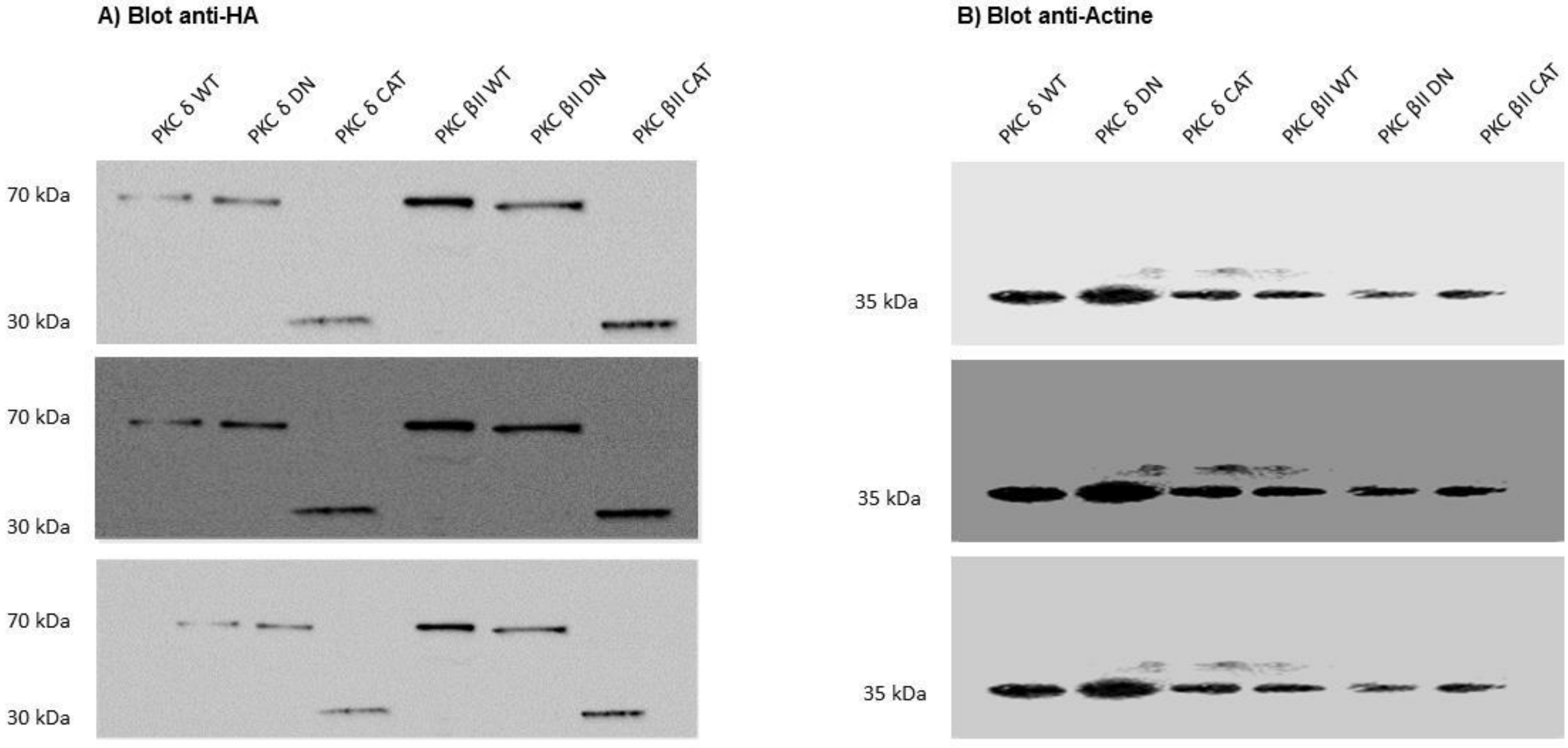

Supplementary Figure 2: Blot anti-HA and anti-Actine: (**A**) full-length blots anti-HA and (**B**) anti-Actine related to figure 3 B. Three exposures are shown.

Supplementary Figure 3

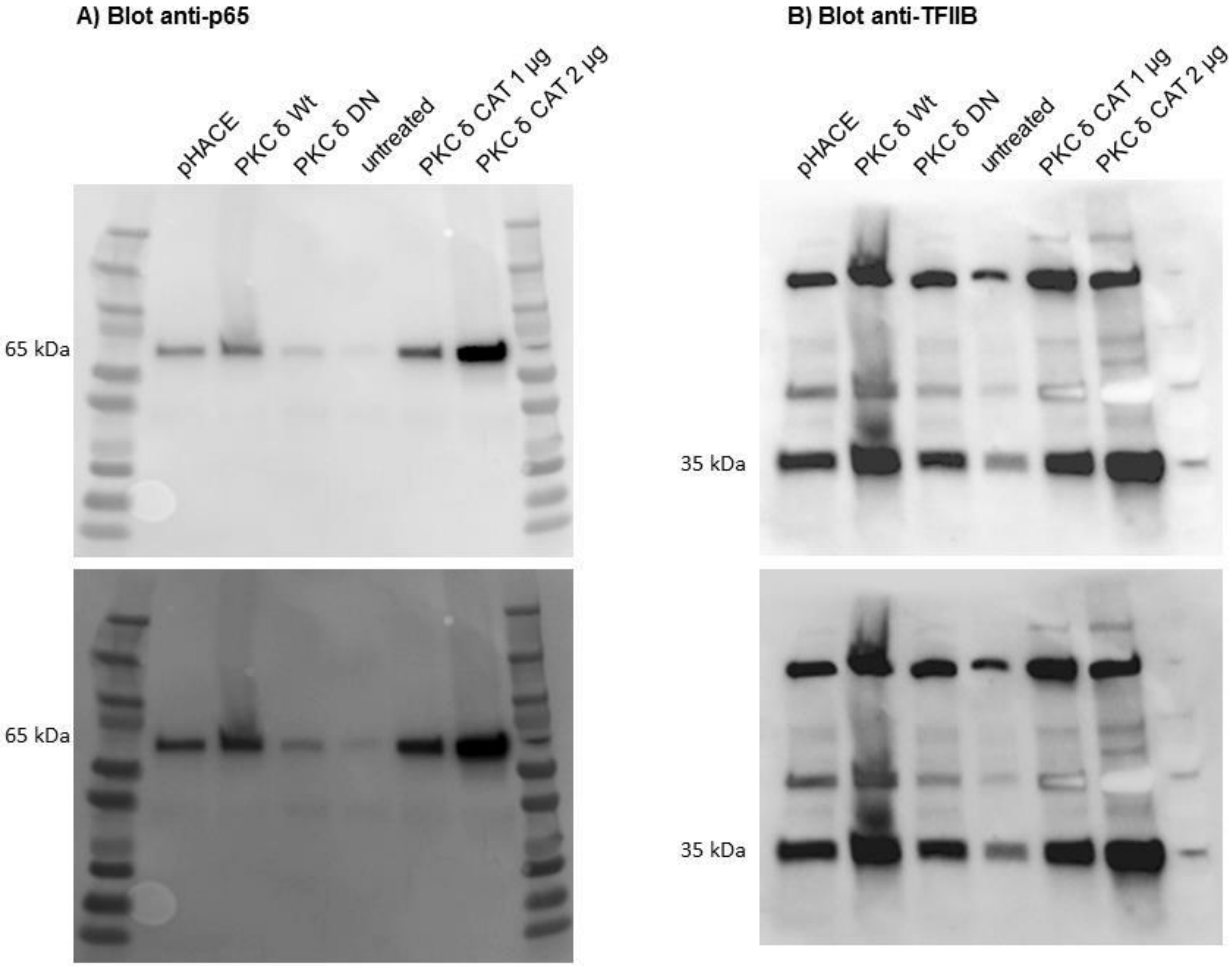

Supplementary Figure 3: Blot anti-p65 and anti-TFFIIB: (**A**) full-length blots anti-p65 and (**B**) anti-TFIIB related to figure 6 A. Two exposures are shown.

Supplementary Figure 4

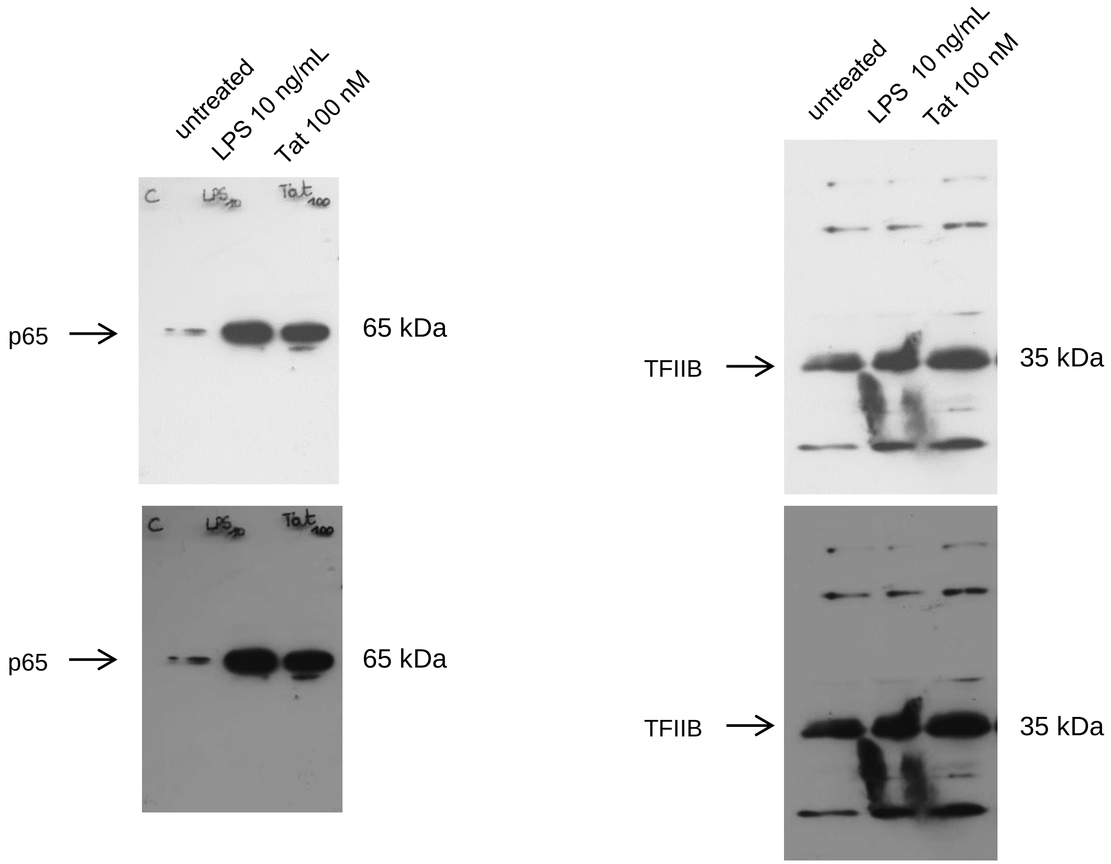

Supplementary Figure 4: Blot anti-p65 and anti-TFFIIB: (**A**) full-length blots anti-p65 and (**B**) anti-TFIIB related to figure 6 B. Two exposures are shown.
